# Elevated CO_2_ enhanced water use efficiency of wheat to progressive drought stress but not on maize

**DOI:** 10.3389/fpls.2022.953712

**Published:** 2022-11-17

**Authors:** Qingjun Cao, Gang Li, Fulai Liu

**Affiliations:** ^1^ Key Laboratory of Northeast crop physiology ecology and cultivation, Ministry of Agriculture and Rural Affairs of The People’s Republic of China, Jilin Academy of Agriculture Science, Changchun, China; ^2^ Department of Plant and Environmental Sciences, Faculty of Science, University of Copenhagen, Taastrup, Denmark

**Keywords:** abscisic acid (ABA), climate change, elevated CO2, gas exchange, stomatal conductance

## Abstract

Global rising atmospheric CO_2_ concentration ([CO_2_]) and drought stress exert profound influences on crop growth and yield. The objective of the present study was to investigate the responses of leaf gas exchange and plant water use efficiency (WUE) of wheat (C3) and maize (C4) plants to progressive drought stress under ambient (a[CO_2_], 400 ppm) and elevated (e[CO_2_], 800 ppm) atmospheric CO_2_ concentrations. The fraction of transpirable soil water (FTSW) was used to evaluate soil water status in the pots. Under non-drought stress, e[CO_2_] increased the net photosynthetic rate (A_n_) solely in wheat, and dry matter accumulation (DMA), whereas it decreased stomatal conductance (*g*_s_) and water consumption (WC), resulting in enhanced WUE by 27.82% for maize and 49.86% for wheat. After onset of progressive soil drying, maize plants in e[CO_2_] showed lower FTSW thresholds than wheat, at which e.g. *g_s_* (0.31 *vs* 0.40) and leaf relative water content (0.21 *vs* 0.43) starts to decrease, indicating e[CO_2_] conferred a greater drought resistance in maize. Under the combination of e[CO_2_] and drought stress, enhanced WUE was solely found in wheat, which is mainly associated with increased DMA and unaffected WC. These varied responses of leaf gas exchange and WUE between the two species to combined drought and e[CO_2_] suggest that specific water management strategies should be developed to optimize crop WUE for different species in a future drier and CO_2_-enriched environment.

## Introduction

With global climate change, agricultural systems will be facing a more changeable environment, such as the rising atmospheric CO2 levels ([CO2]) and more intense and frequent drought events (Lara and Andreo, 2011). According to IPCC projections, the annual rising rate of [CO2] has been accelerated from approximately 0.6 ppm (parts per million)/yr in the 1960 s to about 2.3 ppm/yr (2009-2018), and atmospheric [CO2] will continue to rise from currently 411.29 ppm to 800 ppm by 2100 (Kadam et al., 2014). Thus, field crops probably face the situation with elevated [CO2] (e[CO2]) together with drought stress occurring simultaneously in future climate. Thus, a better knowledge about how plants respond to drought stress under e[CO2] is required for sustainable crop productivity.

Accumulated evidence has shown e[CO_2_] exerts profound impacts on plant growth, phenotypic plasticity, and physiological metabolism (Leakey et al., 2009; Abebe et al., 2016). It has been well documented that plants grow under e[CO_2_] directly stimulate photosynthetic CO_2_ fixation by the increase of Rubisco carboxylation rates and decreasing rates of photorespiration (Kaminski et al., 2014), thus benefiting plant biomass accumulation, productivity, and grain yield (Leakey et al., 2009; Li et al., 2019a), despite acclimation of photosynthetic capacity is observed. However, owing to the variation of CO_2_-fixation efficiency between C3 and C4 species, it seems that the enhancement of carbon assimilation is more beneficial in C3 plants than these C4 plants in e[CO2] illustrated by a meta-analysis in a total of 124 plant species (Zhang et al., 2018). However, differential responses to e[CO2] between genotypes (Lecain et al., 2003), CO2 levels (Hao et al., 2016), and their interactions (Fang et al., 2019) have also been noticed. On the other hand, the negative effect of e[CO2] on plant growth has also been observed in recent studies, e.g. altering the rhizosphere environment by changing the quantity and composition of root exudates and limiting the N bioavailability for plant uptake (Feng et al., 2015); reducing the mass flow of nutrients through the soil to plant due to decreased stomatal conductance and transpiration (Wang et al., 2018). Obviously, these effects caused by e[CO_2_] would modulate the carbon and nitrogen metabolism in plant, which may consequently affect WUE. Yet, the underlying mechanisms influencing WUE between C3 and C4 species under e[CO_2_] remain largely elusive and merit further studies.

Nowadays, the impact of drought stress on crop growth and yield becomes more significant due to global environmental change (Liu et al., 2005; Vialet-Chabrand and Lawson, 2020). It has been well documented that drought stress could induce a range of morphological, physiological transcriptome and cellular changes in plants (Farooq et al., 2009), e.g. cause leaf stomatal closure, reduce plant root and leaf water relations, decrease plant gas exchange rate, or change stomatal behavior, thus directly or indirectly affect plant WUE (Aditi Gupta and Caño-Delgado, 2020). While for a long-term, drought induced adaptation responses include reducing stomatal densities, inhibiting plant growth and development, or even leading to plant death (Arnao and Hernandez-Ruiz, 2014; Shen et al., 2016). Therefore, the negative impacts of drought stress generally depend on the severity of stress and the plant growth stage, as well as plant species and genotypes (Li et al., 2022).

Recent evidence shows that plants respond to multiple stresses differently from how they do to individual stresses (Fan et al., 2020). Earlier studies have demonstrated that multiple drought and e [CO_2_] could reduce the stomatal conductance by modulating stomatal morphology and behavior, thereby decreasing leaf gas-exchange rates while enhancing instantaneous WUE in short term (Tubiello and Ewert, 2002; Haworth et al., 2016). It is well known that the drought-induced stomatal closure is closely linked to the levels of leaf signaling substance-abscisic acid (ABA) (Davies et al., 2002; Liu and Stutzel, 2002). While in a long term, e[CO_2_] and drought stress could decrease stomatal density, and alter the water balance of plants, sap flow and intrinsic water use efficiency, where both ABA signal and leaf turgor are involved and play critical role in regulation those responses (Wei et al., 2022).

At the leaf scale, WUE is primarily related to CO_2_ fixed in photosynthesis (A_n_) and the amounts of water loss from the leaf interior to the atmosphere (Bertolino et al., 2019). More evidences showed both drought stress and e[CO_2_] had profound effects on plant stomata morphology (Zhu et al., 2018a), such as guard cell shape, stomatal density, and stomatal size, all of which relate to stomata behavior, and consequently influence plant A_n_, *g_s_* and plant WUE (Birami et al., 2020; Jalakas et al., 2021). Leaf stomata, which primarily regulated by guard cells plays a key role in regulating water use and carbon assimilation (Buckley, 2019). In drought stressed environment, guard cells on plant leaf can directly sense the reduced hydraulic conductivity and increased [ABA] in xylem cavitation (He et al., 2019), thus decrease guard cell turgor pressure, result in the decease of stomatal pore aperture and *g_s_*, which in turn decrease the rates of CO_2_ uptake for carbon assimilation and water loss, and ultimately influence WUE (Ainsworth and Rogers, 2007; Hao et al., 2016). e[CO_2_] can cause increase in A_n_, result in partial stomata closure in short term and reductions of stomatal density in long term, thereby decreasing *g_s_* and evapotranspiration, thus benefiting for improving plant WUE (Damour et al., 2010; Knipfer et al., 2020). However, the varied physiological and morphological response to [CO_2_] between specific plant species were observed. More evidences showed e[CO_2_] exhibited a great enhancement of A_n_ and obvious reduction for *g_s_* in C3 plants, thus contributed to an obvious increase in WUE (Ullah et al., 2019; Avila et al., 2020). While in C4 plants, e[CO_2_] has little or even no effects on A_n_, and lower deduction in *g_s_*, thus results in a distinct response of WUE from C3 plants in theory (Yang et al., 2022). While plant WUE in canopy level is also associated with the plant leaf area, which directly influence plant transpiration rate (Gao and Tian, 2019). Evidences demonstrated e[CO2] plants usually have a larger leaf area, therefore leading to an increase of water loss (Conley et al., 2001), which in turn may offset the improved plant WUE in e[CO_2_](Henry et al., 2019). Recent studies have observed C3 plants grown in e[CO_2_] had a larger leaf area index increment than those C4 plants (Kirschbaum and Mcmillan, 2018; Qi et al., 2019). Therefore, whether the plant WUE in the combination of drought and e[CO_2_] condition is enhanced is still in doubt.

To investigate how plant gas exchange and WUE of whole plant level responded to e[CO_2_] and long-term drought stress, a progressive soil drying experiment with two plant species of wheat (C3) and maize was conducted under two CO_2_ levels of 400 ppm and 800 ppm in the climatic controlled greenhouse. We hypothesized that the change of A_n_, *g_s_* and plant canopy leaf area, as well as biomass, are not synchronously responded to e[CO_2_], which may contribute to a variation in water consumption and WUE.

## Materials and method

### Experimental design and growth conditions

Seeds of wheat (cv. Gladius) and a wild type of ABA-deficient maize (Vp5) (provided by the Maize Genetics and Genomics Database, USA (MaizeGDB) were used in this study.

This study was conducted at the University of Copenhagen (Taastrup Campus), Denmark, on July 2, 2020. The potted plants were all grown in a temperature-controlled greenhouse. Seeds were placed in a 4 liter pot (diameter 15.2 cm and 25 cm deep) filled with 2.4 kg peat (Plugg-och Såjord Dry matter approximately 80 kg m^-3^, organic matter > 95%, EC 2.5-3.5 ms cm^-1^, pH 5.5-6.5). In total, 96 pots were set up. Eight grains of wheat and two grains of corn after surface disinfected were planted in each pot, and tap water was used for irrigation. After seeding, half of the pots were placed into a glasshouse cell with ambient CO_2_ concentration (400 ppm, a[CO_2_]), and the other half were placed in another glasshouse cell with high CO_2_ concentration (800 ppm, e[CO_2_]).

**Figure 1:**
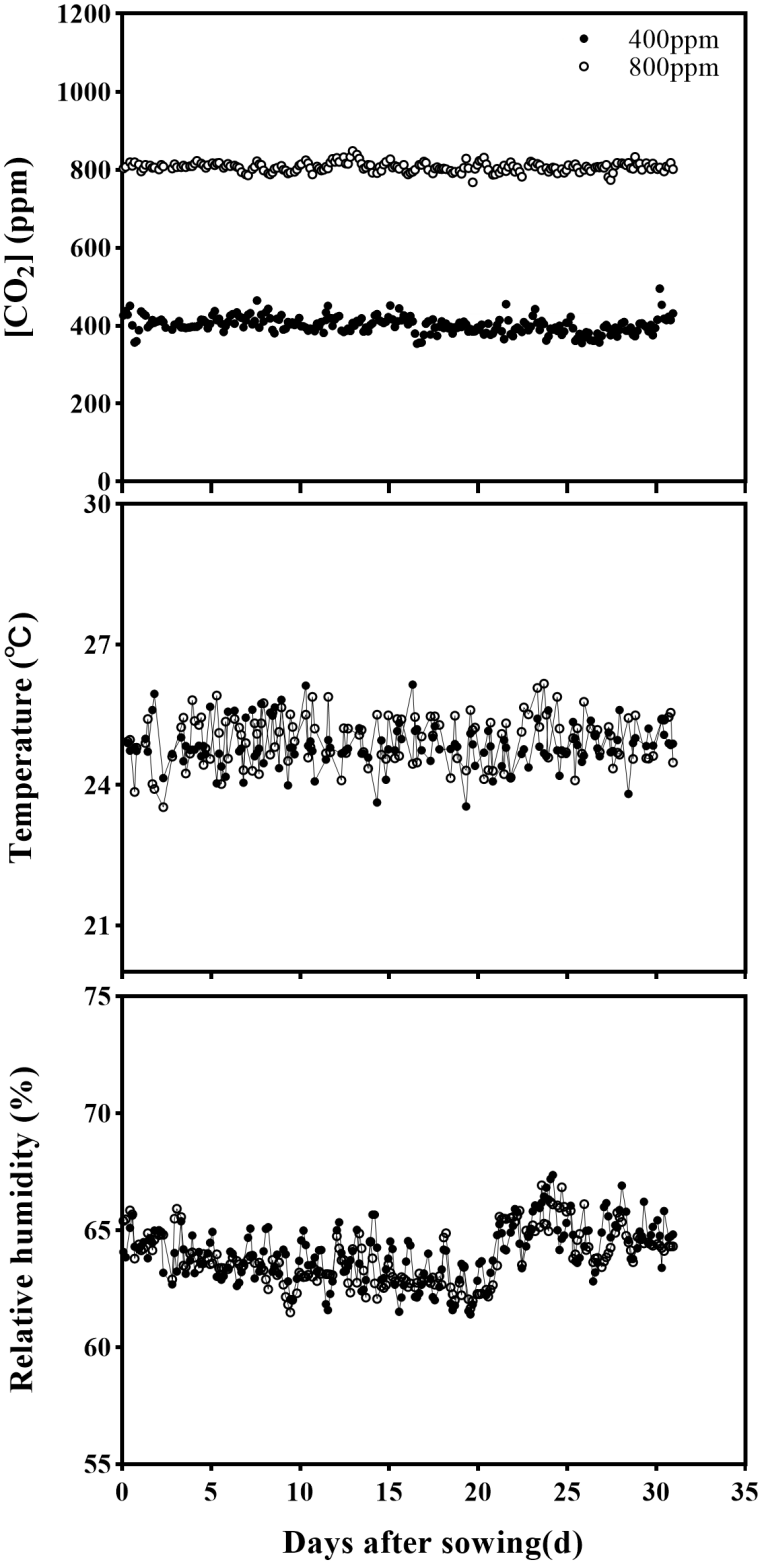
The average of [CO_2_] concentration, temperature (T), and relative humidity (RH) in greenhouse cells designated at 400 and 800 ppm during the experimental period.

In each cell, 96 pots (48 wheat and 48 corn) were randomly distributed on a growth panel, and the CO_2_ was enriched inside the cell *via* emitting pure CO_2_ at one point from a bottle holder and distributed through the ventilation system. The ambient [CO_2_] was monitored every 6 seconds using a CO_2_ transmitter (GMT220 series, Vaisala Group, Helsinki, Finland). Climate conditions in two greenhouse cells were placed at: 25/16 ± 2°C (day/night) air temperature, relative humidity of 65 ± 2.5%, photoperiod of 16 h, and photosynthetic active radiation (PAR) at > 250 μmol m^-2^ s^-1^ is provided *via* sunlight plus LED lamps (**Figure 1**). Climate data is monitored every five minutes periods and recorded by the climate computer. Daily mean [CO_2_], relative humidity, and air temperature in the greenhouse cells throughout the experimental period are presented in **Figure 1**. Two weeks after sowing, corn and wheat plants were thinned in two for wheat and one for maize in each pot, and applied 1.0 g N, 0.7 g P, and 0.8 g K each pot to avoid nutrient deficiency.

**Figure 2:**
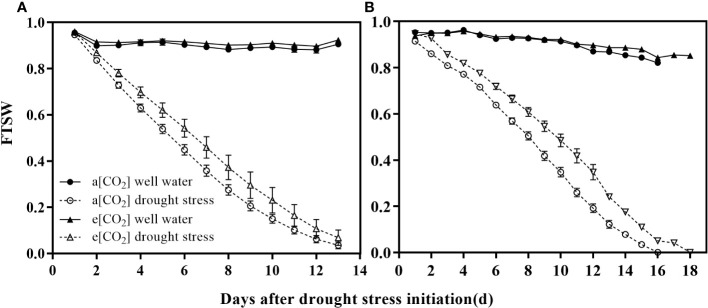
Trends of the fraction of transpirable soil water (FTSW) over time for well-watered and drought-stressed pots in which **(A)** wheat and **(B)** maize were grown under ambient (400 ppm, a [CO_2_]) and elevated (800 ppm, e [CO_2_]) atmospheric CO_2_ concentrations during progressive soil drying. Error bars indicate the standard error of the means (S.E.) (n = 4).

After 2 weeks, half of the wheat and maize seedlings in each cell were subjected to progressive soil drying, and the remaining pots were irrigated daily to maintain 95% water holding capacity (WHC) as the control. The water content in the pot was expressed as the fraction of water in the soil that can transpire (FTSW). Total transpirable soil water (TTSW) is the difference between pot weight at 95% WHC (approximate pot weight 3.5 kg) and when the transpiration rate of the stressed plants is reduces to 10% WHC for with control plants. The daily value of FTSW is estimated as the ratio of the amount of transpiration remaining soil water in the pot to the TTSW:


$$\text{FTSW}=\frac{WT_{n}-WT_{f}}{TTSW}$$

where WT_n_ is the actual pot weight at a given day and WT_f_ is the pot weight at the time when the transpiration rate of stressed plants is 10% compared with control plants (pot weight is about 3.1 kg). The actual pot weight of the pots is obtained by weighing the pots daily during the drying cycle, and the daily transpiration is also determined. The changes in the FTSW during the experiment are shown in **Figure 2**.

### Sampling and measurement

After water stress, net photosynthetic rate (A_n_) and stomatal conductance (*g_s_*) were measured daily on newly fully developed leaves above the canopy leaf from 9:00 am to 11:00 am using the LI-6400 *XT* handheld Photosynthesis System (LiCor Inc., Lincoln, NE, USA). During continuous soil drying, plants from each treatment were harvested five times in different soil water status; and for each species at each harvest, eight plants (4 well-watered plants, WW; and 4 drought-stressed plants, DS) were harvested, and leaf water potential (Ψ_Leaf_) was measured with a pressure chamber (Soil Moisture Equipment Corp., SantaBarbara, CA, USA) on fully developed canopy leaves at 10:00 AM - 11:00 AM, and the rest of the leaf were immediately used for leaf relative water content determination (LRWC) and leaf ABA concentration ([ABA]_leaf_). The LRWC=[(FW-DW)/(SW-DW) × 100%, where FW, DW, SW indicate the fresh weight, dry weight, and saturated weight of the leaf. The [ABA]_leaf_ was determined following the method of Liu et al., 2005. Total leaf area (LA) was measured with a LI-3100 acreage meter (Li-Cor, inc. Lincoln, Nebraska USA), and shoot dry mass (DM) was determined after 72 h of oven drying at 75°C.

### Statistical analysis

Data were analyzed statistically using Microsoft Excel and SPSS 16.0 software (SPSS Inc., Chicago, IL, USA). The effects of water stress (water), ambient CO_2_ concentration (CO_2_), and their interactions (water × CO_2_) on the variables were analyzed using analysis of variance (ANOVA). The difierences between treatments was considered significant when the P-value was less than 0.05 in the Tukey’s HSD test.

The responses of A_n_, g_s_, Ψ_leaf_, and LRWC to soil drying were described using a linear plateau model (Faralli et al., 2019):

If FTSW > C; y=y_initial_

If FTSW < C, y= y_initial_

+S×(FTSW-C) where y means A_n_, *g_s_*, Ψ_leaf_ and LRWC, and y_initial_ means An_max_, g_max_ Ψ_max_ and LRWC_max_, respectively; C is the FTSW threshold at which y begins to deviate from the y_initial_ for A_n_, *g_s_*, Ψ_leaf_ and LRWC (denoted as C_A_, C_g_, C_Ψ_ and C_LRWC_, respectively). Parameters y and C were estimated by PROC NLIN of PC SAS 9.4 (SAS Institute Inc., Cary, NC, USA, 2002-2012) and the coefficient of determination (r^2^) was calculated. Statistical comparison of each parameter obtained from the linear-plateau regression between treatments of [CO_2_] or plant species was performed by t-test using MedCalcstatistical 19.0.7 software.

The relationship between increment of *g_s_* during progressive soil drying and leaf [ABA] was evaluated by linear regression. R^2^ of the regression lines were calculated and the statistical differences on the slopes of the regression lines between *g_s_* and leaf [ABA] in wheat and corn plants under a[CO_2_] and e[CO_2_] were performed separately by analysis of covariance (ANCOVA, WU as covariate).

## Results

### Net photosynthetic rate (A_n_) and stomatal conductance (*g_s_*)

**Figure 3:**
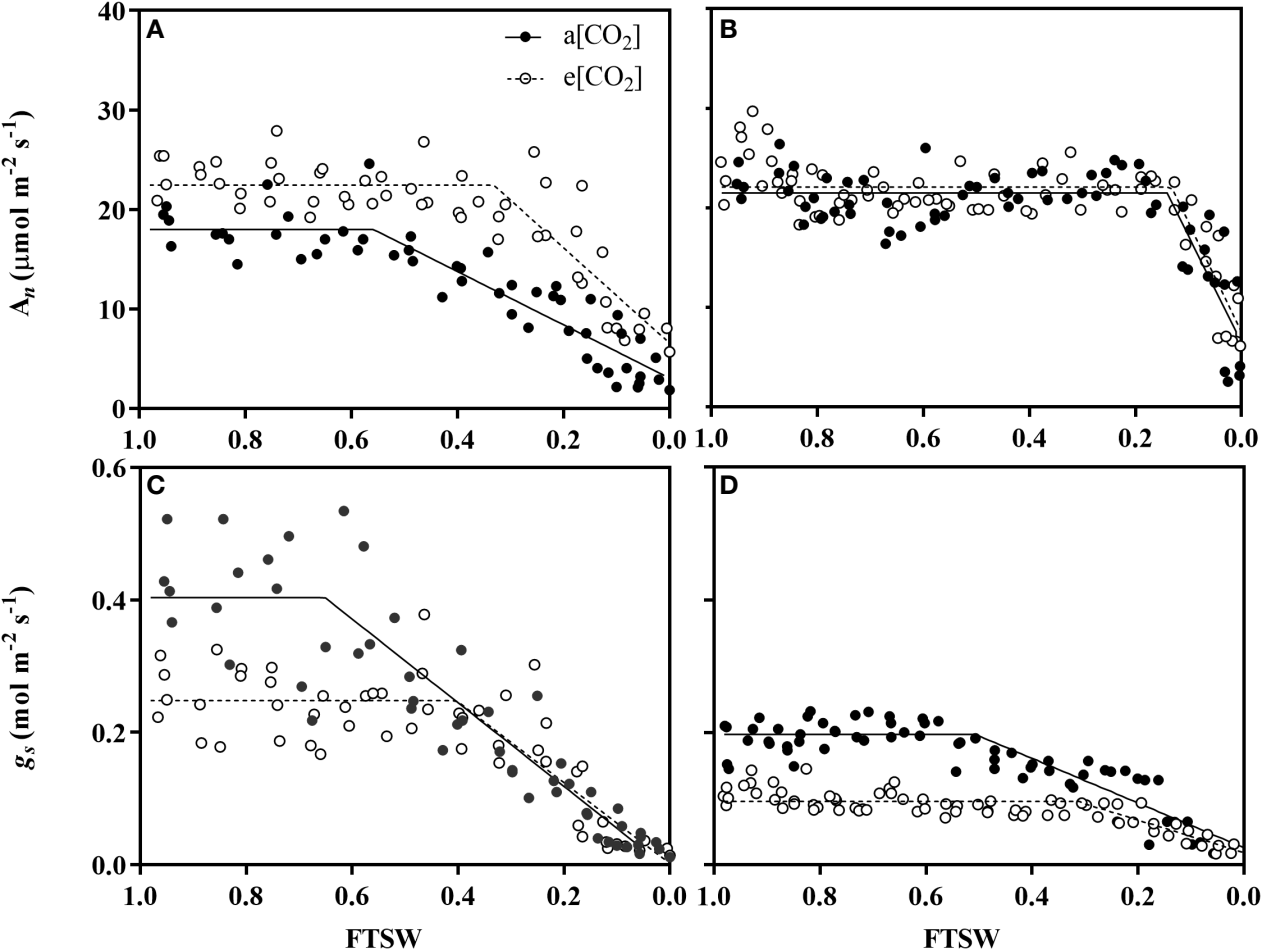
Changes of net photosynthesis rate (A_n_) of **(A)** wheat and **(B)** maize plants and stomatal conductance (*g_s_*) of **(C)** wheat and **(D)** maize plants grown under ambient (400 ppm, a[CO_2_]) and elevated (800 ppm, e[CO_2_]) atmospheric CO_2_ concentrations during progressive soil drying. Closed and open circles indicate plants at a[CO_2_] and e[CO_2_] concentration, respectively. Error bars indicate standard error of the means (SE) (n = 4).

The change of net photosynthetic rate (A_n_) and stomatal conductance (*g_s_*) under a[CO_2_] and e[CO_2_] were shown in **Figure 3**. Under non-stressed condition, A_n_ of wheat plant grown under e[CO_2_] was 22.46 μmol m^-2^ s^-1^ and 24.91% higher than those grown under a[CO_2_] (**Figure 3A**), while no differences for maize (**Figure 3B**). During progressive soil drying, A_n_ under e[CO_2_] started to decline at a significant lower FTSW threshold (Cg) than that under a[CO_2_] (i.e., 0.33 vs 0.56) (**Figure 3A**; **Table 1**).

**Table 1:** Significant test for linear-plateau model parameters of net photosynthetic rate (A_n_), stomatal conductance (*g_s_*), leaf water potential (ψ_Leaf_), and relative leaf water content (RLWC).

Genotype	[CO_2_]	A_n_		*g_s_*		ψ_Leaf_		LRWC	
		A_n max_	C_A_	*g*_s max_	C_g_	ψ_max_	C_ψ_	LRWC_max_	C_LRWC_
Wheat	400 ppm	17.98	0.56	0.40	0.65	-1.17	0.47	87.87	0.54
	800 ppm	22.46	0.33	0.25	0.40	-1.28	0.38	91.17	0.43
	Sig.	**	**	**	**	ns	*	ns	*
Maize	400 ppm	21.48	0.32	0.20	0.50	-0.58	0.29	93.91	0.24
	800 ppm	22.08	0.19	0.096	0.31	-0.61	0.25	92.64	0.21
	Sig.	**ns**	*	**	*	**ns**	**ns**	**ns**	**ns**

**Figure 4:**
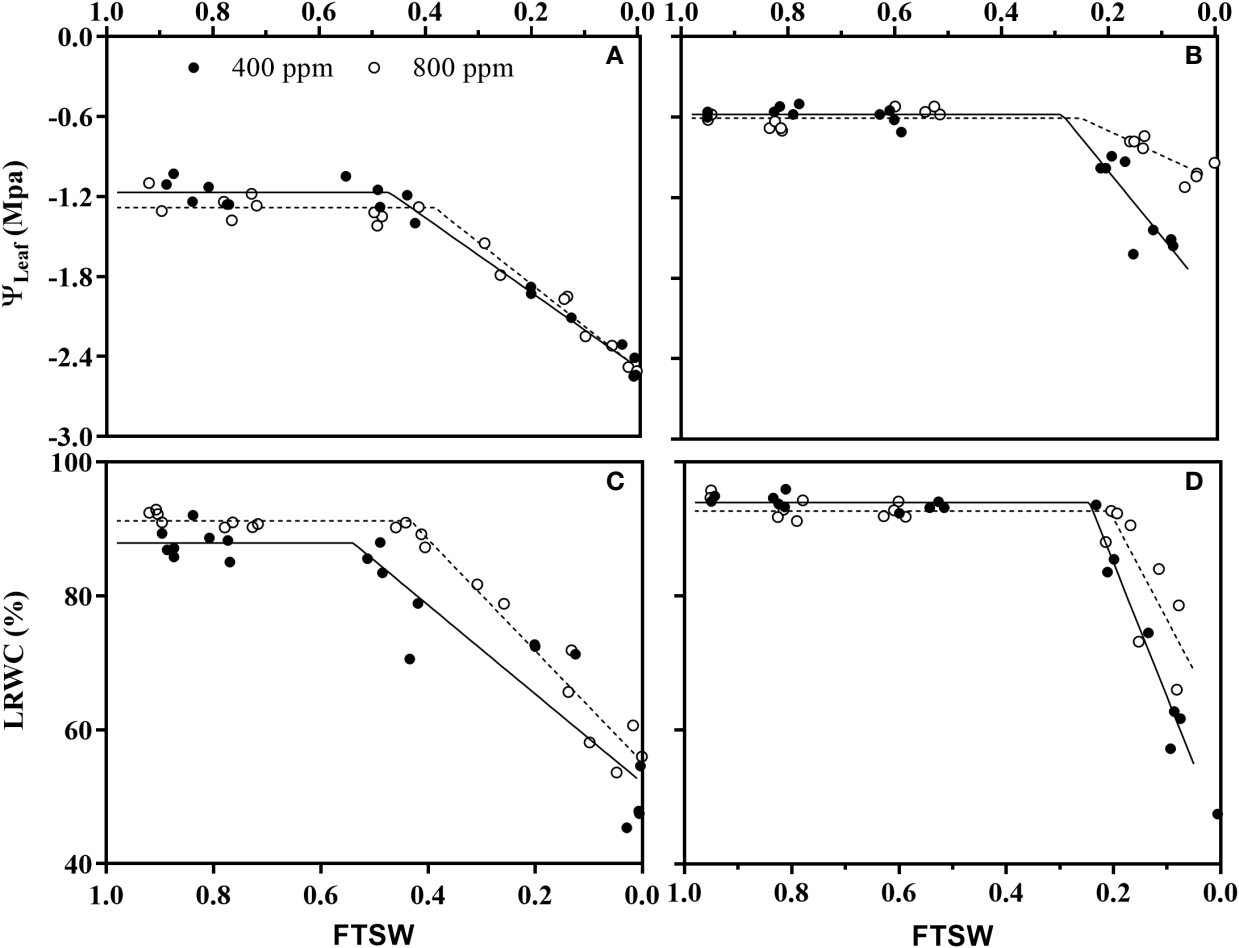
Changes of leaf water potential (ψ_Leaf_) of **(A)** wheat and **(B)** maize plants, and relative leaf water content (RLWC) of **(C)** wheat and **(D)** maize plants grown under ambient (400 ppm, a[CO_2_]) and elevated (800 ppm, e[CO_2_]) atmospheric CO_2_ concentrations during progressive soil drying. Closed and open circles indicate plants at a[CO_2_] and e[CO_2_] concentration, respectively.

A_n max_, g_s max_, ψ_max_ and LRWC _max_, indicated the initial values of the parameters when the plants were not significantly affected by drought; C (C_A_, C_g,_ C_ψ_ or C_LRWC_) indicated the threshold at which the parameter (A_n_ or g_s_, respectively) start to decrease due to drought stress. The data is presented in **Figures 3** and **4**. * and ** indicate differences significant at 0.05 and 0.01 levels; ns, not significant.

During the initiation time of drought stress, both wheat and maize plant grown under e[CO_2_] had lower *g_s_* value than those grown under a[CO_2_](**Figures 3C, D**). *g_s_* under e[CO_2_] started to decline at a significant lower FTSW threshold (C_g_) than that under a[CO_2_] (i.e., 0.40 vs 0.65 for wheat, and 0.31 vs 0.50 for maize) during progressive soil drying (**Figures 3C, D**; **Table 1**). The *g_s_*_max_ of wheat plants were 0.25 μmol m^-2^ s^-1^ and 37.5% lower than that plants grown under a[CO_2_], and *g_s_*_max_ of maize plants were 0.096 μmol m^-2^ s^-1^ and decreased by 52.0% (**Table 1**), correspondingly.

### Leaf water relation

During the initiation time of drought stress, no significance differences of ψ_max_ and LRWC_max_ were observed under e[CO_2_] and a[CO_2_] for either wheat nor maize plants (**Figure 4**; **Table 1**). During the progressive soil drying, e[CO_2_] plants showed significant lower C_ψ_ (**Figure 4A**; **Table 1**) and C_LRWC_ (**Figure 4C**; **Table 1**) than those a[CO_2_] plants for wheat, while no differences between a[CO_2_] and e[CO_2_] for maize (**Figures 4B, D**; **Table 1**).

### Leaf area

**Figure 5:**
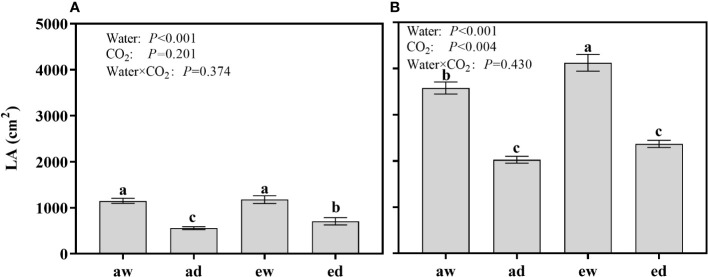
Changes of leaf area (LA) of **(A)** wheat and **(B)** maize plants grown under ambient (400 ppm, a[CO_2_]) and elevated (800 ppm, e[CO_2_]) atmospheric CO_2_ concentrations during progressive soil drying. Error bars indicate standard error of the means (SE) (n = 4). The different small letters among treatments in the figure means differences significant at the 0.01 level.

For wheat, LA was significantly affected by drought stress (Water), while not affected by CO2 concentration (CO_2_) and the interaction (Water × CO_2_). Under DS, the LA of e[CO_2_] plants 27.09% higher than a[CO_2_] plants (**Figure 5A**), while no different between e[CO_2_] and a[CO_2_] under WW condition.

For maize, LA was significantly affected by DS and environmental CO_2_ (**Figure 5B**). DS plants had lower LA as expected, while e[CO_2_] showed an opposite effect. Under WW condition, LA of e[CO_2_] plants increased 15.22% than a[CO_2_] plants, while differences were not significant under DS.

### Water consumption, dry matter accumulation and water use efficiency

**Figure 6:**
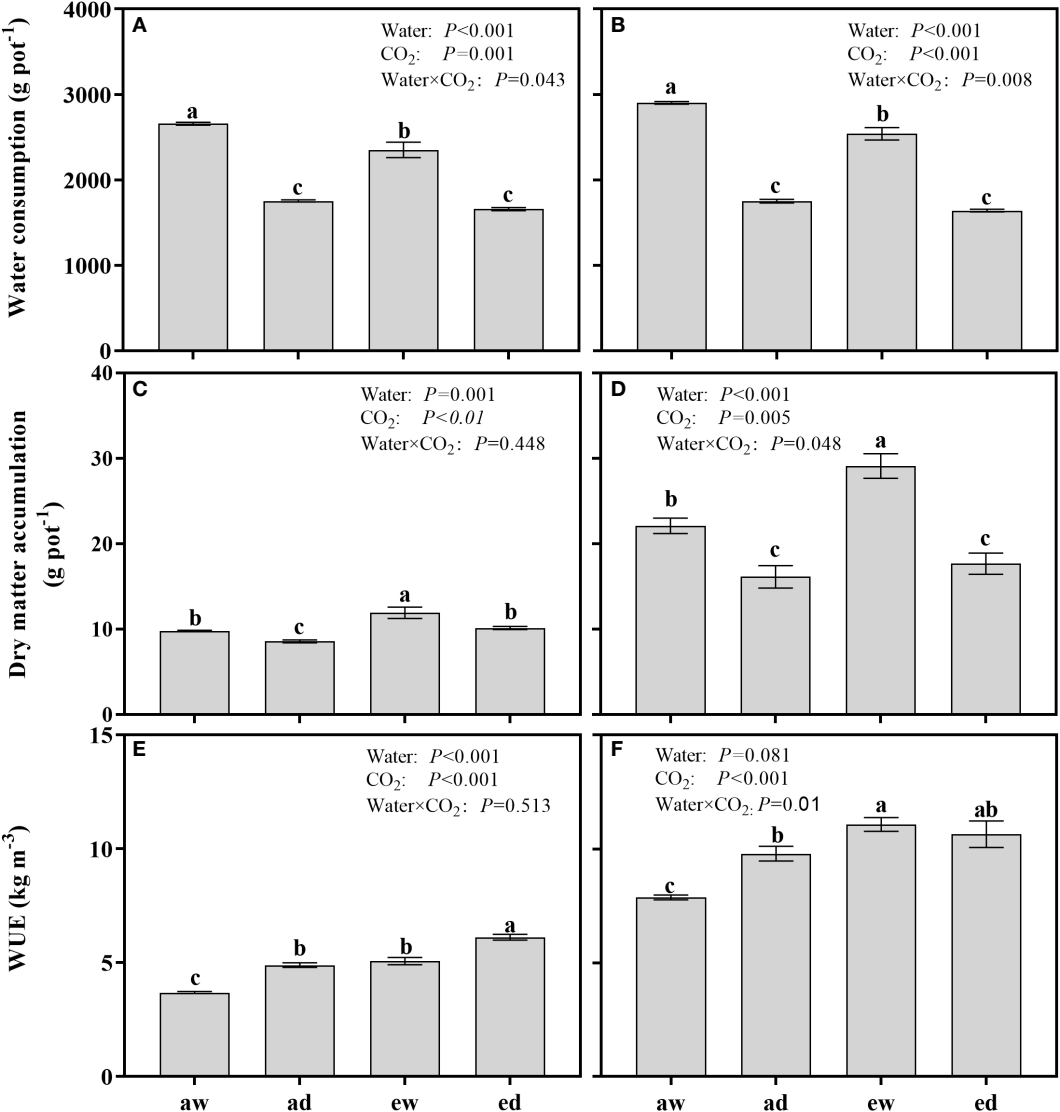
Comparison of water consumption (WC), dry matter accumulation(DMA), and water use efficiency (WUE) of wheat **(A, C, E)** and maize **(B, D, F)** plants grown under ambient (400 ppm, a[CO_2_]) and elevated (800 ppm, e[CO_2_]) atmospheric CO_2_ concentrations during progressive soil drying. Error bars indicate standard error of the means (SE) (n = 4). The different small letters among treatments in the figure means differences significant at the 0.01 level.

The WC and DMA from the initiation of drought stress to final sample harvest were found significant affected by both DS and environmental CO_2_ (**Figures 6A–D**). For WC, both wheat and maize under WW in e[CO_2_] showed significantly higher WC compared with a[CO_2_] plants (**Figures 6A, B**), while no differences were observed between e[CO_2_]and a[CO_2_] plants under DS. For DAM, e[CO_2_] enhanced DMA under both WW and DS condition for wheat plants (**Figure 6C**), whereas the difference of DMA for maize only observed under WW condition in e[CO_2_] plants (**Figure 6D**).

For WUE, both DS and e[CO_2_] significant enhanced WUE in wheat plant (**Figure 6E**). Compared to WW plants, WUE of a[CO_2_] and e[CO_2_] were enhanced by 32.83% and 22.55% under DS, respectively. In maize plants, (**Figure 6F**) the WUE were significantly affected by environmental CO_2_ and the interaction of DS and environmental CO_2_ (Water × CO_2_). Compared to a[CO_2_], e[CO_2_] plants under WW and DS condition were enhanced by 39.29% and 8.74%, respectively.

### Leaf ABA concentration during progressive drought stress

**Figure 7:**
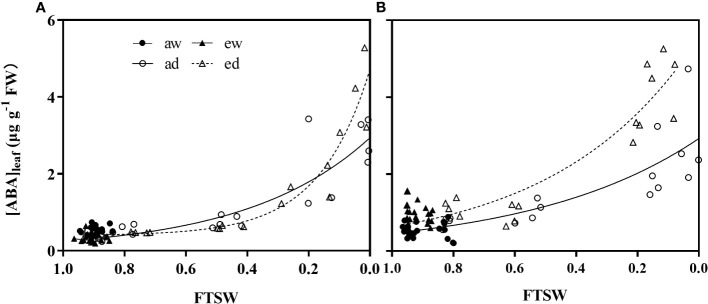
Changes of leaf ABA concentration of **(A)** wheat and **(B)** maize plants under ambient (400 ppm, a[CO_2_ ]) and elevated (800 ppm, e[CO_2_ ]) atmospheric CO_2_ concentrations during progressive soil drying.

**Figure 8:**
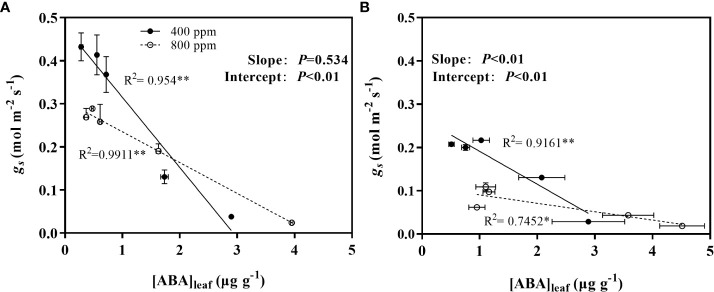
Relations between leaf ABA concentration ([ABA]leaf) with stomatal conductance (*g_s_*)of wheat **(A)** and maize **(B)** plants grown under ambient (400 ppm) and elevated (800 ppm) atmospheric CO_2_ concentrations during progressive soil drying. Error bars indicate standard error of the means (SE) (n = 4).

DS and environmental CO_2_ significant affected the ABA concentration in leaf ([ABA]_leaf_) (**Figure 7**). For WW plants (aw and ew), [ABA]_leaf_ in both wheat (**Figure 8A**) and maize plant (**Figure 8B**) had lower value, while showed an opposite trend in DS (ad and ed). [ABA]_leaf_ in DS increased exponentially during progressive soil drying for both wheat and maize plants grown under both CO_2_ environments. In addition, for maize plant, e[CO_2_] showed higher [ABA]_leaf_ relative to the a[CO_2_] plants (**Figure 7B**), while significant higher [ABA]_leaf_ only be observed during the final stage for wheat plant.

To further understand how *g_s_* response to [ABA]_leaf_ during the progressive soil drying, the linear regression analysis were conducted and presented in **Figures 8A, B**. *g_s_* decreased linearly with the increase of [ABA]_leaf_ for both plant genotypes and environmental CO_2_, and the intercepts of the regression lines were significantly differed between the two CO_2_ environments for both plants, while significant differences of the slope of the regression lines between a[CO_2_] and e[CO_2_] was only observed for maize plants.

## Discussion

Atmospheric Carbon dioxide (CO_2_) is an essential substrate for photosynthesis, due to the diffidence of the CO_2_-concentrating mechanism between C3 and C4 plants (Kadam et al., 2014; Liu et al., 2019), plants with C3 photosynthetic pathway grown in e[CO_2_] mostly exhibit greater enhancement of A_n_ than those with a C4 pathway (Prior et al., 2011). Consistent with this, wheat plants in e[CO_2_] treatment under the non-drought stress conditions, exhibited an obvious enhancement of A_n_ than those grown in a[CO_2_] (**Figure 3A**), whereas no differences were observed in maize plants (**Figure 3B**). This varied response of A_n_ to e[CO_2_] between wheat plant and maize plant could be attributed to the CO_2_ concentrate in the bundle sheath cell, elevated CO_2_ concentration can induce C3 plants to intake more CO_2_ molecules thus stimulating leaf photosynthesis (Allen et al., 2011; Liu et al., 2019). Moreover, during the progressive soil drying, the FTSW threshold of A_n_ for the wheat plant in e[CO_2_] is 0.33, which is significantly lower than that of in a[CO_2_] with 0.56. The retarded responses of A_n_ to soil drying under e[CO_2_] for wheat plants again confirmed e[CO_2_] that induced plant drought adaption, and similar results have been confirmed by the recent study of Yang et al., 2022 in tomato, as well as Wei et al., 2022 in amaranth and maize.

Stomata plays a central role in controlling plant CO_2_ and H_2_O exchange between the interior of leaf and the exterior environment (Lawson, 2009), which is very sensitive to the exterior environment, such as environmental [CO_2_] and water status changes (Mcainsh and Taylor, 2017; Zhu et al., 2018a). In the present study, it was observed that the *g_s_* in both plant species were lower in e[CO_2_], compared to a[CO_2_], the results are in agreement with the common conclusion that e[CO_2_] could decrease the *g_s_* by modulating the stomatal aperture in a short term effect. Interestingly, compared to maize, a more pronounced reduction of *g_s_*_max_ was found in wheat plants (**Figures 3C, D**), This is in agreement with the common conclusion that plants grown in e[CO_2_] generally caused a great reduction in *g_s_* for C3 plants than C4 species, which also indicates that C4 plant may have a higher environmental [CO_2_] tolerance than C3 plants in environmental climate change.

Generally, e[CO_2_] reduces leaf *g_s_* and consequently regulates CO_2_ and H_2_O exchange between plants and the atmosphere, thus influencing leaf water relations (Qiu et al., 2008; Birami et al., 2020). In the present study, e[CO_2_] had no effect on leaf water potential (ψ_Leaf_) and LRWC during the onset of the drought stress (**Figure 4**). The similar results were also found in tomato (Wei et al., 2018), wheat and other plant species (Li et al., 2019b), and this could be attributed to plant’s morphological and physiological adaptation strategies to conserve leaf water balance for survival, such as decreasing transpiration rate by stomatal regulation (Chavan et al., 2019), enhancing photosynthetic enzyme activities for more non-structural carbohydrate concentration (e.g. sucrose, hexose, starch), and higher water use efficiency (Liu et al., 2004; Li et al., 2019b). Additionally, under drought stress condition, the threshold of LRWC and ψ_Leaf_ for both species in e[CO_2_] were significantly lower than those plants grown in a[CO_2_], which indicates the plants grown in e [CO_2_] retarded the response of plants to soil water deficit. And this e[CO_2_]-induced response is possibly associated with “hydroactive feedback”, which involved abscisic acid (ABA) production in plant leaves (Liu et al., 2003; Li et al., 2020).

Stomatal regulation also plays an important role in influencing plant growth and water consumption (Mcainsh and Taylor, 2017). Early studies illustrated that e[CO_2_] could decrease the *g_s_* by modulating the stomatal movement (e.g. stomatal aperture (SA) in a short term or changing stomatal morphology (e.g. stomatal size and stomatal density (SD) in a long time (Xu et al., 2016; Mcainsh and Taylor, 2017). Hence, the combination of e[CO_2_] and drought-induced stomata response could reduce the *g_s_* and subsequent leaf water status, thereby decreasing water consumption by modulating plant morphology, physiology, and biochemistry response (Qiu et al., 2008; Birami et al., 2020). As expected, under non-drought stress condition, both species at e[CO_2_] showed lower water consumption than those plants grown in a[CO_2_], the positive linear relationship between water consumption and *g_s_* (figure not showed) indicating that the water consumption is primarily related to the *g_s_* changes.

While on the whole-plant level, the plant water consumption is not only related to *g_s_* changes, but is also associated with the stomatal morphology (Peters et al., 2018), and factors that influence the crop canopy evapotranspiration (Xu et al., 2016), such as leaf area, leaf temperature, vapour pressure deficit (VPD) in a long-term effect (Jalakas et al., 2021). Evidences also showed that the “trade-offs” effect between reductions in stomatal conductance and increases in leaf area are critical to plant water consumption (Berens et al., 2019). Here, the water consumption of both species between treatments ad and ed were not significant (**Figures 6A, B**), the result indicating the progressive water deficit may diminish the e[CO_2_] induced water conservation effect due to increment of LA, together with subsequent water transpiration in wheat. This phenomenon was also confirmed in soybean using the free air CO_2_ enrichment method (Bernacchi et al., 2007) and other studies (Hatfield and Dold, 2019). Further, studies noticed that e[CO_2_] could impair the effectiveness of stomatal closure (Haworth et al., 2016), thereby weakened the conservation-effect induced by e[CO_2_] and resulting in inevitable water consumption to severe water deficit, thus contributing to the insignificant water consumption between treatment ad and ed for both species.

In recent years, the fraction of transpirable soil water (FTSW) is widely used to evaluate the soil water status in drought stress (Liu and Stutzel, 2002; Yang et al., 2022). In the present study, the leaf gas exchange parameters (A_n_ and *g_s_*), as well as leaf water relations (ψ_Leaf_ and LRWC) to progressive soil water deficit were estimated. It was found that both wheat and maize plants are grown in e[CO_2_] showed lower A_n_, *g_s_*, ψ_Leaf_ and LRWC FTSW thresholds (except A_n_ and LRWC for maize) values (**Figures 3** and **4**, **Table 1**), suggesting that e[CO_2_] modulates the plant drought stress adoption. Meanwhile, FTSW thresholds of maize in DS under e[CO_2_] were also significantly lower than those grown in maize (eg. *g_s_* was 0.33 for wheat vs 0.19 for maize). The results indicate that the maize plants may become less sensitive to soil drying when exposed to e[CO_2_]. The varied response of plant genotypes or species to plant gas exchange parameters on An and *g_s_* were also consistent with the earlier studies by (M, 2015; Abebe et al., 2016) in maize and by Liu et al., 2019 in the tomato plants. The latest research also confirms our findings that the different response to drought stress and e[CO_2_] were also existed between C4 species amaranth (dicot) and maize (monocot) (Wei et al., 2022), although the mechanisms are still unclear.

For C3 plants, e[CO_2_] stimulate An leading to a CO_2_-fertilizing effect, thereby increasing plant growth and subsequently increased plant biomass (Lecain et al., 2003), while little or even no effects on C4 plants, such as maize and sorghum (Wang et al., 2012). Here, it was found that both wheat and maize grown in e[CO_2_] showed obvious increase in LA and DMA under non-drought stress (**Figures 5**, **6B, C**), as well as maize plants under drought stress (**Figures 5B** and **6C**), while the effect was not significant for maize in drought stress. This might be due to various adaptation strategies. Earlier studies showed that e[CO_2_] can regulate more carbon allocation to root, thus altering root or shoot architecture, e.g. LA, root-shoot ratio (Wullschleger et al., 2002; Zhu et al., 2018b). For maize plants, e[CO_2_] might regulate more production of fine roots to gather scarce water for progressive soil drying. However, more evidence in root traits should be investigated.

Plant WUE is an early response indicator for evaluating the physiological or ecological response to environmental change (Hatfield and Dold, 2019). In most cases, e[CO_2_] could improve the leaf-level WUE due to the decreased leaf transpiration rate by moderating *g_s_*, while no effect on leaf photosynthesis under mild and moderate drought stress (Oliveira et al., 2016; Zhang et al., 2018), while for a long-term effect, evidence showed that that drought stress has a stronger impact on *g_s_* than e [CO_2_] (Ullah et al., 2019; Li et al., 2020), thus result in a complex response. In present studies, under progressive drying, wheat plants grown in e[CO_2_] accelerated An than those in a[CO_2_], hereby increase DMA (**Figure 6C**), and further contribute to a greater WUE. While for maize plants, e[CO_2_] combined drought stress showed no effect on DMA (**Figure 6D**), due to neither DMA nor water consumption was affected by e[CO_2_] to progressive water status. Recent evidence shows that plants respond to multiple stresses differently from how they do to individual stresses (Berens et al., 2019), our results provided direct evidence that C3 plants and C4 plants might have different modulation mechanisms to e[CO_2_] and progressive water deficit. Thus, specific water management strategies should be developed to optimize crop WUE in a future global atmospheric drier and CO_2_-enriched.

To cope with the diverse stress conditions, plants evolved and developed various adaptation strategies response, these responses to different stresses are highly complex and may be involved in the changes at the transcriptome, cellular, and physiological levels (Vanaja et al., 2015). Stomatal closure is regarded be an essential strategy to defend against the combination of e[CO_2_] and drought stress. Increasing evidence showed that soil drying-induced stomatal closure is mainly regulated by the root-to-shoot ABA signal (Liu et al., 2005), and leaf turgor during severe drought (Jalakas et al., 2021). Consistent with this, the leaf ABA concentration of both species was found significantly enhanced during the final stage of drought stress (**Figure 7**), which confirm that the leaf ABA could act as a signal substance to participate in plants’ drought adaptation in e[CO_2_]. Additionally, the effect of e[CO_2_] on plant growth and performance is rather complex, except for its directly effect on plant carbon metabolism, but also related to air temperature by trapping heat effect (Dusenge et al., 2019). Thus, more attention about the interaction effect of e[CO_2_] and air temperature on plant species response should be paid in the future.

## Conclusion

During the progressive soil drying, e[CO_2_] of both wheat and maize species exhibited lower FTSW threshold of An, *g_s_*, ψ_Leaf_ and LRWC than grown in a[CO_2_], indicating that e[CO_2_] modulate a greater plant drought stress adaptation; meanwhile, the gradually increased leaf [ABA], together with its negative linear relationship with *g_s_* were observed in both species, suggesting leaf ABA may play important role in the combination of e[CO_2_] and progressive drying modulated drought adaptation. To progressive soil drying, e[CO_2_] showed higher DMA, but no effect on LA and WC, thus attributed to higher WUE in wheat, while not affected maize WUE. Conclusively, the varied responses of leaf gas exchange and WUE to e[CO_2_] and soil water deficits in wheat and maize species might be due to different modulation mechanisms. Therefore, specific water management strategies should be developed to optimize crop WUE in a future global atmospheric drier and CO_2_-enriched.

## Data availability statement

The original contributions presented in the study are included in the article/**Supplementary Material**. Further inquiries can be directed to the corresponding author.

## Author contributions

FL designed the experiments. QC conducted the experiments and analyzed the data. QC wrote the manuscript with the help of GL and FL. All authors contributed to the article and approved the submitted version.

## Funding

This work was financially supported by the Agricultural science and technology innovation project of Jilin Province CXGC201903GH.

## Acknowledgments

This is a short text to acknowledge the contributions of specific colleagues, institutions, or agencies that aided the efforts of the authors.

## Conflict of interest

The authors declare that the research was conducted in the absence of any commercial or financial relationships that could be construed as a potential conflict of interest.

## Publisher’s note

All claims expressed in this article are solely those of the authors and do not necessarily represent those of their affiliated organizations, or those of the publisher, the editors and the reviewers. Any product that may be evaluated in this article, or claim that may be made by its manufacturer, is not guaranteed or endorsed by the publisher.

## Abbreviations

e[CO_2_], elevated atmospheric CO_2_ concentrations; a[CO_2_], ambient atmospheric CO_2_ concentrations; A_n_, net photosynthetic rate; *g*_s_, stomatal conductance; Ψ_Leaf_, leaf water potential; LRWC, leaf relative water content; DMA, dry matter accumulation; WHC, water holding capacity; WUE, water use efficiency.
