# Supplementary material for: Elevated CO2 enhanced water use efficiency of wheat to progressive drought stress but not on maize
Source: Front Plant Sci. 2022 Nov 17;13:953712. doi: 10.3389/fpls.2022.953712 (PMC9714360; doi:10.3389/fpls.2022.953712)
Supplement: Supplementary file 1 [file DataSheet_1.pdf]

Meteorological data of greenhouse cell 1 during the experiment

Days after stress initiation, d	Celle 1.Temperature, °C	Celle 1.RH, %	Celle 1.[CO2], ppm
**0.06**	17.95	70.48	17.95
**0.19**	18.05	73.67	18.05
**0.31**	22.89	73.25	18.49
**0.44**	24.73	61.91	18.33
**0.56**	24.88	58.40	18.48
**0.69**	24.80	57.83	18.40
**0.81**	24.73	58.93	18.33
**0.94**	21.86	63.23	17.86
**1.06**	18.04	69.73	18.04
**1.19**	18.12	73.21	18.12
**1.31**	22.98	68.20	18.98
**1.44**	24.71	58.72	18.71
**1.56**	24.42	60.82	18.42
**1.69**	23.60	67.14	17.60
**1.81**	23.95	65.08	17.95
**1.94**	21.65	64.78	18.65
**2.06**	18.01	70.97	19.01
**2.19**	18.14	74.52	18.14
**2.31**	22.15	71.43	18.15
**2.44**	23.28	69.73	18.48
**2.81**	24.66	64.88	19.16
**2.94**	21.66	66.13	18.66
**3.06**	17.92	70.01	17.92
**3.19**	17.97	72.39	17.97
**3.31**	22.01	70.58	18.01
**3.44**	24.51	69.31	18.51
**3.56**	24.83	60.33	18.83
**3.69**	24.70	56.99	18.70
**3.81**	24.75	57.96	18.47
**3.94**	24.75	58.28	18.75
**4.06**	24.75	58.28	18.75
**4.19**	24.75	58.28	18.75
**4.31**	23.84	63.22	19.04
**4.44**	24.61	60.58	18.61
**4.56**	24.83	58.52	18.13
**4.69**	24.66	59.00	18.66
**4.81**	24.01	60.49	18.01
**4.94**	21.67	62.44	18.67
**5.06**	17.95	68.67	17.95
**5.19**	17.38	72.23	18.38
**5.31**	22.04	67.24	18.04
**5.44**	24.67	58.37	18.67
**5.56**	24.40	58.06	18.40
**5.69**	23.69	60.64	18.69
**5.81**	23.17	61.50	18.17
**5.94**	21.56	60.56	18.56
**6.06**	16.74	64.90	18.74
**6.19**	16.34	67.12	18.34
**6.31**	22.58	63.29	18.58
**6.44**	24.78	58.37	18.78
**6.56**	24.72	57.33	18.72
**6.69**	24.78	57.13	18.78
**6.81**	24.05	59.58	18.05
**6.94**	21.44	60.56	18.44

**7.06**	17.06	65.14	18.61
**7.19**	16.23	67.39	18.63
**7.31**	22.61	64.09	18.61
**7.44**	24.61	58.46	18.61
**7.56**	24.68	58.15	18.68
**7.69**	24.77	59.77	18.77
**7.81**	23.73	61.80	18.73
**7.94**	21.47	61.15	18.47
**8.06**	16.70	64.28	18.70
**8.19**	16.46	66.56	18.46
**8.31**	21.54	69.09	18.54
**8.44**	21.48	55.65	18.48
**8.56**	20.65	54.50	18.65
**8.69**	24.05	59.49	19.05
**8.81**	24.36	60.08	19.36
**8.94**	20.82	62.43	18.82
**9.06**	17.26	67.80	18.26
**9.19**	16.63	70.71	19.63
**9.31**	22.39	65.01	18.39
**9.44**	24.80	58.88	18.80
**9.56**	24.80	57.61	18.80
**9.69**	24.65	57.96	18.65
**9.81**	23.78	59.54	17.78
**9.94**	21.61	61.42	18.61
**10.06**	17.13	64.81	20.13
**10.19**	16.48	67.93	20.48
**10.31**	22.12	65.87	21.12
**10.44**	24.84	59.13	21.84
**10.56**	24.93	57.39	21.93
**10.69**	24.73	58.56	21.73
**10.81**	24.08	62.23	22.08
**10.94**	21.57	62.40	21.57
**11.06**	17.81	67.10	22.81
**11.19**	16.70	70.20	22.70
**11.31**	21.77	68.99	22.77
**11.44**	24.54	61.84	23.54
**11.56**	24.96	57.28	23.96
**11.69**	24.80	57.26	23.80
**11.81**	24.78	59.44	24.78
**11.94**	21.62	61.50	24.62
**12.06**	17.74	66.40	24.74
**12.19**	17.50	71.34	24.50
**12.31**	22.67	67.26	24.67
**12.44**	24.69	58.34	24.69
**12.56**	24.74	57.68	24.74
**12.69**	24.77	59.31	24.77
**12.81**	24.69	64.19	24.69
**12.94**	21.63	62.67	23.63
**13.06**	17.96	69.53	22.96
**13.19**	18.27	73.35	23.27
**13.31**	22.21	69.66	24.21
**13.44**	24.67	58.34	24.67
**13.56**	24.67	59.16	24.67
**13.69**	24.62	58.63	24.62
**13.81**	24.58	59.73	24.58
**13.94**	21.63	61.93	24.63
**14.06**	17.90	68.05	23.90
**14.19**	17.63	72.74	25.63

**14.31**	22.62	68.54	25.62
**14.44**	24.92	58.99	24.12
**14.56**	24.95	57.80	24.95
**14.69**	24.76	59.04	24.76
**14.81**	24.11	63.23	24.11
**14.94**	21.76	64.14	24.76
**15.06**	18.17	70.51	24.17
**15.19**	17.95	73.58	23.95
**15.31**	22.74	68.05	24.74
**15.44**	25.15	58.57	25.15
**15.56**	25.32	56.40	25.32
**15.69**	24.98	57.44	24.18
**15.81**	24.67	58.60	24.67
**15.94**	21.65	62.74	25.65
**16.06**	17.92	67.79	24.92
**16.19**	17.35	71.45	24.35
**16.31**	23.14	67.26	25.14
**16.44**	25.15	58.45	25.15
**16.56**	25.19	57.36	25.19
**16.69**	24.89	57.62	24.89
**16.81**	24.74	62.52	24.74
**16.94**	21.63	62.69	23.63
**17.06**	17.93	68.69	24.93
**17.19**	17.98	71.48	24.98
**17.31**	22.51	65.97	24.51
**17.44**	25.01	59.05	25.01
**17.56**	25.26	57.60	25.26
**17.69**	24.86	58.23	24.86
**17.81**	24.76	59.16	24.76
**17.94**	21.59	61.27	24.59
**18.06**	17.91	67.36	24.91
**18.19**	17.99	71.32	24.99
**18.31**	21.82	68.54	24.82
**18.44**	24.77	60.71	24.77
**18.56**	25.02	58.15	25.02
**18.69**	24.87	57.80	24.87
**18.81**	24.82	59.62	24.82
**18.94**	21.72	61.78	25.72
**19.06**	17.76	66.67	25.76
**19.19**	16.83	69.74	24.83
**19.31**	22.54	66.89	24.54
**19.44**	24.99	59.68	24.99
**19.56**	25.10	57.56	25.10
**19.69**	24.87	58.00	24.87
**19.81**	24.41	60.09	24.41
**19.94**	21.72	62.01	26.72
**20.06**	17.71	66.36	25.71
**20.19**	16.64	68.65	24.64
**20.31**	22.69	65.80	24.69
**20.44**	24.95	59.71	24.95
**20.56**	25.16	57.52	25.16
**20.69**	24.82	58.29	24.82
**20.81**	24.07	61.81	25.07
**20.94**	21.89	62.03	23.89
**21.06**	17.59	66.28	25.73
**21.19**	16.44	69.28	24.44
**21.31**	22.40	67.35	24.40
**21.44**	24.96	60.00	24.96

**21.56**	24.79	59.96	24.79
**21.69**	24.74	60.94	24.74
**21.81**	24.14	65.78	24.14
**21.94**	21.70	63.64	24.70
**22.06**	17.99	70.39	25.99
**22.19**	18.15	74.19	25.15
**22.31**	21.63	71.02	25.63
**22.44**	24.66	65.53	25.66
**22.56**	24.76	60.52	24.76
**22.69**	24.82	59.36	24.82
**22.81**	24.37	64.92	24.37
**22.94**	21.73	63.67	23.73
**23.06**	17.74	68.67	25.74
**23.19**	16.65	70.80	24.65
**23.31**	22.42	69.17	24.42
**23.44**	24.81	60.59	24.81
**23.56**	24.68	60.53	24.68
**23.69**	24.68	60.43	24.68
**23.81**	24.61	64.93	24.61
**23.94**	21.59	63.52	24.59
**24.06**	17.94	68.26	24.94
**24.19**	17.97	71.13	24.97
**24.31**	22.03	71.20	24.03
**24.44**	24.74	68.09	24.74
**24.56**	24.20	70.75	25.20
**24.69**	24.74	62.81	24.74
**24.81**	24.65	63.29	24.65
**24.94**	21.66	64.25	25.66
**25.06**	18.00	69.81	25.00
**25.19**	17.11	72.34	24.61
**25.31**	22.35	69.88	25.35
**25.44**	24.99	59.70	24.99
**25.56**	24.85	59.14	24.85
**25.69**	24.77	59.51	24.77
**25.81**	24.49	65.58	24.49
**25.94**	21.63	64.21	24.63
**26.06**	17.93	68.62	25.93
**26.19**	17.98	71.72	24.98
**26.31**	22.38	69.71	24.38
**26.44**	24.88	60.02	24.88
**26.56**	25.04	58.48	24.04
**26.69**	24.78	58.97	24.78
**26.81**	24.61	61.28	24.61
**26.94**	21.72	62.65	24.72
**27.06**	17.99	67.43	24.99
**27.19**	17.31	70.87	24.31
**27.31**	22.12	68.79	25.12
**27.44**	24.69	60.60	24.69
**27.56**	24.72	59.04	24.72
**27.69**	24.37	63.82	24.37
**27.81**	23.66	62.62	24.66
**27.94**	21.60	63.09	24.60
**28.06**	18.05	70.10	25.05
**28.19**	17.67	73.46	25.67
**28.31**	21.40	69.13	24.80
**28.44**	23.81	65.31	24.81
**28.56**	24.68	59.77	24.68
**28.69**	24.89	59.68	24.89

**28.81**	24.00	65.10	25.00
**28.94**	21.70	62.83	24.70
**29.06**	18.27	70.16	24.27
**29.19**	18.08	73.52	25.08
**29.31**	21.86	70.92	24.86
**29.44**	24.83	61.47	24.83
**29.56**	25.20	56.98	25.20
**29.69**	25.00	57.27	25.00
**29.81**	24.84	59.80	24.84
**29.94**	21.65	62.24	24.65
**30.06**	17.88	67.08	25.38
**30.19**	16.79	69.92	24.79
**30.31**	22.40	68.31	25.40
**30.44**	25.07	58.20	24.07
**30.56**	25.17	56.86	24.17
**30.69**	24.89	57.56	24.89
**30.81**	24.85	60.64	24.85
**30.93**	21.88	60.82	24.88

Meteorological data of greenhouse cell 2 during the experiment

Days after stress initiation, d	Celle 1.Temperature, °C	Celle 1.RH, %	Celle 1.[CO2], ppm
**0.06**	25.41	79.12	795.21
**0.19**	24.02	78.12	811.21
**0.30**	24.16	78.63	800.73
**0.31**	24.93	77.46	803.64
**0.44**	24.96	75.19	817.54
**0.56**	24.34	77.80	819.58
**0.69**	23.85	78.57	810.05
**0.81**	24.79	76.57	816.73
**1.31**	24.89	79.12	813.37
**1.44**	25.41	79.12	795.21
**1.56**	24.02	79.30	817.57
**1.69**	24.02	78.12	811.21
**1.81**	23.91	76.17	804.96
**2.31**	23.52	79.68	805.45
**2.44**	24.16	78.63	800.73
**2.81**	24.61	76.50	812.23
**3.31**	25.23	80.09	797.73
**3.44**	25.44	80.09	803.63
**3.56**	24.25	76.81	814.64
**3.69**	24.26	78.24	806.44
**3.81**	24.67	80.60	818.12
**3.94**	25.81	81.01	806.53
**4.06**	25.37	81.01	810.60
**4.19**	24.08	79.26	806.92
**4.31**	25.28	76.10	809.64
**4.44**	25.45	79.86	809.24
**4.56**	24.43	81.04	809.01
**4.69**	24.78	81.04	816.24
**4.81**	24.32	78.14	822.03
**4.94**	24.55	77.18	816.14
**5.31**	25.91	80.33	811.67
**5.44**	25.11	80.47	805.23
**5.56**	24.02	80.47	811.49
**5.69**	25.86	80.47	816.97
**5.81**	25.35	78.81	812.07
**5.94**	24.56	76.90	816.84
**6.31**	25.41	80.74	811.47
**6.44**	25.17	80.74	810.27
**6.56**	25.23	78.39	815.14
**6.69**	25.07	80.84	809.23
**6.81**	24.32	81.60	807.24
**6.94**	24.89	80.56	794.07
**7.31**	24.30	80.56	798.77
**7.44**	25.32	80.56	787.73
**7.56**	25.09	80.56	785.36
**7.69**	24.23	80.56	785.36
**7.81**	25.32	80.56	801.80
**7.94**	25.75	80.56	807.42
**8.31**	24.65	80.56	821.97
**8.44**	25.54	80.20	814.55
**8.56**	24.81	79.29	812.45
**8.69**	24.16	81.58	797.91
**8.81**	25.13	81.84	784.21
**8.94**	25.65	81.84	790.28
**9.31**	24.52	80.89	793.05

**9.44**	25.51	77.44	801.98
**9.56**	25.24	81.36	811.96
**9.69**	24.93	77.58	810.32
**9.81**	25.17	78.62	800.24
**10.31**	25.50	77.63	798.03
**10.44**	24.59	77.64	790.68
**10.56**	24.81	78.80	792.78
**10.69**	25.88	77.43	793.64
**10.81**	25.20	76.43	795.05
**11.44**	24.68	75.36	800.48
**11.56**	25.88	75.36	811.04
**11.69**	24.70	75.35	814.62
**11.81**	25.00	74.09	824.61
**12.31**	24.10	73.40	824.06
**12.44**	25.21	74.19	788.06
**12.56**	24.68	73.24	823.76
**12.69**	25.20	74.31	798.36
**12.81**	24.67	74.31	799.44
**13.31**	25.07	74.31	807.56
**13.44**	25.18	74.31	831.53
**13.56**	24.70	74.30	848.17
**13.69**	25.24	73.38	847.45
**13.81**	24.35	78.18	830.03
**14.31**	25.50	77.44	820.13
**14.44**	24.69	73.21	826.00
**14.56**	24.64	73.42	831.75
**14.69**	25.43	75.06	816.16
**14.81**	24.55	74.11	833.26
**14.94**	25.49	74.11	847.80
**15.31**	24.57	74.11	846.68
**15.44**	25.41	72.95	828.70
**15.56**	24.62	72.40	815.22
**15.69**	25.40	73.32	802.48
**15.81**	25.39	71.45	806.03
**16.31**	24.45	67.13	812.43
**16.44**	25.45	70.36	811.51
**16.56**	24.47	70.17	783.14
**16.69**	25.60	64.44	806.67
**16.81**	25.04	62.95	813.43
**17.31**	25.47	61.81	819.88
**17.44**	25.05	66.46	827.28
**17.56**	25.47	63.29	805.44
**17.69**	24.35	61.99	809.80
**17.81**	25.28	60.58	802.29
**18.44**	24.15	62.90	821.57
**18.56**	24.57	65.86	812.94
**18.69**	25.48	64.23	792.82
**18.81**	24.57	60.51	788.40
**19.31**	24.31	62.39	791.96
**19.44**	25.10	64.79	794.51
**19.56**	25.60	61.31	799.85
**19.69**	25.17	64.90	812.38
**19.81**	25.23	62.19	812.86
**20.31**	24.12	62.28	821.14
**20.44**	25.07	65.02	817.51
**20.56**	24.32	62.06	797.07
**20.69**	25.33	64.26	790.89
**20.81**	24.30	63.27	806.66

**20.94**	25.32	64.88	802.06
**21.31**	25.09	61.28	801.18
**21.44**	24.23	62.75	803.66
**21.56**	25.32	65.03	801.50
**21.69**	25.54	64.48	791.04
**21.81**	24.16	63.17	802.39
**22.44**	25.13	64.89	796.77
**22.56**	25.65	61.32	789.93
**22.69**	24.08	61.35	804.94
**22.81**	25.51	64.52	803.78
**23.31**	26.07	59.48	774.46
**23.44**	25.24	57.73	767.90
**23.56**	24.93	61.86	802.74
**23.69**	26.17	62.18	812.53
**23.81**	25.50	59.63	822.82
**23.94**	24.59	59.53	824.17
**24.31**	24.81	63.48	831.42
**24.44**	25.88	62.72	814.29
**24.56**	25.20	59.37	799.67
**24.69**	24.68	60.51	811.89
**24.81**	25.88	62.76	786.85
**24.94**	24.70	59.99	788.07
**25.31**	25.00	62.54	802.60
**25.44**	24.10	62.10	792.32
**25.56**	25.21	62.09	796.65
**25.69**	24.68	59.69	807.37
**25.81**	24.67	62.42	819.00
**25.94**	25.77	62.41	808.82
**26.31**	25.20	59.63	794.42
**26.44**	24.67	61.49	805.56
**26.56**	25.07	63.48	794.39
**26.69**	25.18	61.03	782.25
**26.81**	24.70	60.06	791.55
**27.31**	25.24	62.43	803.99
**27.44**	25.05	60.64	810.24
**27.56**	24.35	61.69	820.76
**27.69**	25.50	62.83	817.45
**27.81**	24.69	60.23	808.08
**27.94**	24.64	62.40	814.80
**28.44**	25.43	63.57	811.63
**28.56**	24.62	60.34	799.21
**28.69**	24.55	62.44	810.91
**28.81**	25.49	63.27	804.31
**29.44**	24.57	60.55	795.04
**29.56**	24.56	62.95	802.73
**29.69**	25.41	63.57	805.63
**29.81**	24.62	60.21	804.06
**30.31**	25.40	62.64	790.84
**30.44**	25.39	61.74	794.52
**30.56**	24.45	61.46	801.63
**30.69**	25.45	63.97	792.05
**30.81**	25.54	62.01	798.93

	Treatment	Daily water consumption during drought stress																	
		1	2	3	4	5	6	7	8	9	10	11	12	13	14	15	16	17	18
	**aW_1**	94.00	82.00	89.00	79.20	105.40	146.10	139.80	148.80	122.20	166.00	201.00	262.00	268.00	305.00	319.60	363.00	non	non
	**aW_2**	99.00	106.00	100.00	67.00	121.90	159.90	151.30	151.50	184.80	185.00	226.70	270.50	285.20	315.00	323.00	359.40	non	non
	**aW_3**	89.00	100.00	94.00	72.00	109.70	138.00	149.10	134.80	159.00	151.00	182.00	229.60	229.90	250.00	271.90	311.00	non	non
	**aW_4**	87.00	103.00	103.00	71.00	129.60	150.00	124.40	150.00	178.80	173.50	205.80	259.00	266.20	279.00	311.80	347.30	non	non
	**aD_1**	110.00	116.00	112.00	81.00	124.30	163.70	142.70	140.20	168.00	132.50	135.10	127.20	89.90	49.40	73.00	41.80	non	non
	**aD_2**	93.00	84.00	88.00	60.00	89.40	136.60	123.30	127.50	154.20	135.00	149.90	161.50	173.80	49.80	68.10	52.80	non	non
	**aD_3**	88.00	87.00	84.00	60.00	89.90	130.10	118.80	116.20	137.00	127.00	139.50	141.90	124.40	107.20	87.00	67.40	non	non
Maize	**aD_4**	103.00	104.00	87.00	70.30	100.00	127.20	115.10	118.60	132.70	114.80	135.70	136.00	116.48	108.42	91.80	84.00	non	non
	**eW_1**	74.00	71.00	66.00	67.70	73.00	77.40	85.90	88.30	101.60	101.80	128.00	138.00	154.80	155.00	173.20	230.90	209.00	208.90
	**eW_2**	82.00	61.00	75.20	67.20	90.00	111.80	99.00	112.90	121.60	127.00	163.20	168.50	180.90	180.00	195.70	247.60	231.40	233.20
	**eW_3**	136.00	84.00	86.30	67.20	100.80	116.20	112.00	121.80	138.50	138.50	163.50	172.60	195.20	184.30	198.40	258.50	230.00	227.50
	**eW_4**	77.00	73.00	70.20	66.80	77.10	94.20	97.90	94.60	114.40	115.00	149.40	155.20	158.20	169.00	175.00	222.50	226.60	238.40
	**ed_1**	76.00	74.00	66.00	50.00	68.00	84.00	81.60	84.20	98.90	86.30	103.50	109.50	116.20	107.60	104.40	107.80	92.00	84.20
	**ed_2**	86.00	83.00	75.00	67.00	74.00	101.00	93.30	96.60	107.80	94.00	115.90	115.40	122.20	101.70	107.70	81.40	77.00	60.50
	**ed_3**	77.00	71.00	66.00	52.00	72.00	89.60	86.20	90.60	96.50	91.50	114.20	112.40	118.20	101.90	101.90	99.00	85.70	73.60
	**ed_4**	85.00	77.00	74.00	58.00	80.40	95.40	94.60	96.60	105.30	130.60	96.20	119.90	124.90	103.70	97.40	91.20	75.50	60.50
	**aW_1**	220.9	219.9	217.1	204.5	199.9	224.1	250.6	275	237.5	247.2	257	256.6	146.5	non	non	non	non	non
	**aW_2**	182.2	181.1	176.3	161.1	154.5	170.8	190.6	210.3	185.7	196.4	203.5	205.91	128.5	non	non	non	non	non
	**aW_3**	164.9	160.2	157.4	149.6	147.6	165.6	185.4	197.4	187.8	201	216.3	217.2	136.5	non	non	non	non	non
	**aW_4**	205.9	205.6	202.3	190.3	184.8	211.3	231.2	250.7	230.6	247.3	265.2	257.3	160.6	non	non	non	non	non
	**aD_1**	219.8	220.6	204.6	171.3	168.2	177.5	162	133.4	96.6	81.3	70	53.2	27.6	non	non	non	non	non
	**aD_2**	196.8	203.2	190.8	175.3	161.5	160.2	144.5	123.3	91.1	79	68.6	53.6	29.1	non	non	non	non	non
	**aD_3**	177	172.4	171.1	157.3	150.1	167.4	172.1	160.4	116.5	106.5	89.9	68.8	34.4	non	non	non	non	non
Wheat	**aD_4**	187.6	186.2	170.1	153.9	142.5	147.1	144.6	137.2	112.5	100.8	89.8	70.4	38.6	non	non	non	non	non
	**eW_1**	161.4	157.1	161.8	149.8	154.4	166.7	173.2	203.2	185.2	184.8	200.7	195.6	116.3	non	non	non	non	non
	**eW_2**	168.1	158.6	160.8	145.4	150.6	168.2	176.2	202.8	178.5	163.9	191.1	173.3	101.7	non	non	non	non	non
	**eW_3**	155	152.7	158.9	154	161.6	182.9	192.8	217.4	178.3	208.1	203.9	213.1	126.2	non	non	non	non	non
	**eW_4**	149.3	143.1	147.1	134.2	137.8	151.1	160.6	179.7	165.7	169.8	174.4	168.4	101	non	non	non	non	non
	**ed_1**	180.6	174.2	171.2	154.2	146.6	160.1	157.1	154.2	115	88.7	85.8	61.8	36.6	non	non	non	non	non
	**ed_2**	139	141.6	135.1	119	112.4	121.8	127.1	149.7	74.8	175.4	136.8	105.5	62.8	non	non	non	non	non
	**ed_3**	141.7	132.2	129.3	114.9	109.2	120.6	124.9	143	126	115.5	147.2	120	68.2	non	non	non	non	non
	**ed_4**	172.5	177.5	181.5	169	163.1	179.5	184.5	187.1	132.8	97.3	87.9	61.6	35.2	non	non	non	non	non

Note: aw= 400 ppm & well-wateded; ad= 400 ppm & drought stressed; ew=800 ppm & well-watered; ed= 800 ppm & droughted stressed.

Net photosynthetic rate (A_n, μmol m^{-2} s^{-1})

	Treatment	Days after stress initiatio (d)																	
		1	2	3	4	5	6	7	8	9	10	11	12	13	14	15	16	17	18
Maize	**aW_1**	27.70	24.30	21.10	20.80	23.00	22.50	21.00	22.90	24.20	27.20	34.20	29.30	32.10	32.10	31.20	30.40	non	non
	**aW_2**	23.10	22.90	25.80	22.20	24.70	22.60	22.80	27.60	20.20	3.20	33.80	28.90	30.20	30.20	30.90	31.50	non	non
	**aW_3**	20.80	21.40	21.70	20.80	21.10	20.20	23.10	21.50	22.40	24.40	29.50	29.00	35.00	33.70	30.80	31.70	non	non
	**aW_4**	23.40	27.40	21.10	19.80	23.60	23.60	20.80	19.50	21.80	26.60	30.20	29.20	32.80	32.60	31.20	32.40	non	non
	**aD_1**	22.10	24.20	23.00	19.40	20.50	18.80	22.10	20.90	20.90	23.50	22.70	19.30	26.10	23.60	12.60	3.51	non	non
	**aD_2**	24.60	26.40	20.10	19.10	20.30	17.60	26.00	21.30	21.50	20.80	27.30	17.80	24.40	23.80	12.30	6.07	non	non
	**aD_3**	22.40	23.50	18.30	18.90	22.60	16.40	18.10	17.20	23.00	26.50	21.20	19.50	24.80	23.10	13.10	2.52	non	non
	**aD_4**	15.90	21.70	21.00	19.60	22.80	17.20	19.40	18.20	20.10	23.70	21.50	20.30	24.30	23.80	12.50	3.14	non	non
	**eW_1**	24.50	28.80	22.10	21.40	22.20	23.90	21.20	21.90	24.60	28.00	27.90	26.20	34.40	33.70	31.50	32.00	31.90	33.80
	**eW_2**	23.00	28.10	20.10	20.90	23.90	19.60	20.10	18.70	21.80	24.20	29.90	26.40	36.10	31.60	32.30	30.90	32.50	37.90
	**eW_3**	25.30	30.40	24.20	21.00	26.20	23.60	20.20	21.20	25.70	24.40	29.40	26.60	31.40	35.80	33.00	29.70	31.80	30.80
	**eW_4**	18.90	28.10	25.50	21.90	22.50	24.40	23.20	21.20	23.70	27.70	28.40	28.30	34.10	33.30	31.80	31.40	32.50	32.60
	**ed_1**	20.10	27.90	23.40	19.10	20.50	17.60	20.90	20.90	19.80	21.20	20.50	19.80	19.60	18.70	16.80	12.20	10.90	6.90
	**ed_2**	20.30	25.40	24.70	18.70	23.90	18.80	21.20	20.20	20.80	24.70	18.40	19.40	16.60	20.80	14.90	10.80	9.60	6.62
	**ed_3**	22.70	29.70	22.60	20.70	23.30	20.80	22.10	20.60	20.20	19.80	19.70	19.90	17.30	20.10	16.60	16.10	12.20	7.07
	**ed_4**	24.60	27.10	22.20	19.50	18.30	19.20	17.30	21.80	19.50	22.50	20.40	19.90	21.30	18.00	17.20	14.30	11.10	6.11
Wheat	**aW_1**	23.3	18.3	19.5	21.7	20.1	17.8	19.9	17.8	17.9	18.5	18.3	18.3	19.4	non	non	non	non	non
	**aW_2**	18.5	19.8	18.3	19.5	18.1	17.7	17.4	19.4	18.9	17.7	18.7	17.8	17.5	non	non	non	non	non
	**aW_3**	20.6	20.6	17.5	18.1	18.2	18.2	18.1	18.7	19.2	19.2	18.8	20.4	20	non	non	non	non	non
	**aW_4**	17.5	18.5	18.3	19	17.6	17.9	19.2	19.5	18.9	19.3	18.9	17.7	17.5	non	non	non	non	non
	**aD_1**	16.3	14.5	15	15.9	15.9	14.1	12.4	12.3	11	9.38	3.21	2.9	1.85	non	non	non	non	non
	**aD_2**	18.9	17	19.3	17.8	15.4	11.2	15.7	8.12	10.9	7.54	3.61	4.06	2.53	non	non	non	non	non
	**aD_3**	19.5	17.5	22.5	15.5	17	17.3	12.8	9.48	11.3	5.02	2.15	7.01	5.1	non	non	non	non	non
	**aD_4**	20.3	17.6	17.5	17	24.6	14.8	14.3	11.6	11.7	7.8	4.04	7.53	2.14	non	non	non	non	non
	**eW_1**	21.9	24.1	22.6	25.4	24.2	22.4	21.5	23.3	27.3	26	19.7	23.6	27.4	non	non	non	non	non
	**eW_2**	22.1	22.4	20.8	25.8	22.5	21.8	22.6	21.4	21.5	21.1	24.2	20.6	21.7	non	non	non	non	non
	**eW_3**	24.1	23.7	23	26	24	24.6	23.3	23.4	25.4	22.2	22.9	22.5	24.8	non	non	non	non	non
	**eW_4**	25.6	26	22.8	28.3	24.9	26.3	26.2	25.3	25.1	20.6	25.7	28.2	24.2	non	non	non	non	non
	**ed_1**	22.5	22.6	24.7	23.7	22.9	22.1	19.7	20.5	22.7	17.8	15.7	6.84	7.96	non	non	non	non	non
	**ed_2**	20.9	24.3	21.6	23.1	20.8	20.5	21.4	20.7	19.2	19.3	17.4	12.6	8.11	non	non	non	non	non
	**ed_3**	25.4	23.5	20.1	27.9	19.2	21.3	23.3	20.5	23.4	17	17.3	13.2	10.7	non	non	non	non	non
	**ed_4**	25.4	24.8	20.8	24.1	20.6	26.8	20.8	25.8	22.4	8.05	9.56	8.06	5.7	non	non	non	non	non

Note: aw= 400 ppm & well-wateded; ad= 400 ppm & drought stressed; ew=800 ppm & well-watered; ed= 800 ppm & droughted stressed.

Leaf stomatal conductance (g_s, μmol m^{-2} s^{-1})

	Treatment	Days after stress initiatio (d)																	
		1	2	3	4	5	6	7	8	9	10	11	12	13	14	15	16	17	18
Maize	**aW_1**	0.17	0.18	0.18	0.23	0.21	0.21	0.16	0.26	0.24	0.25	0.28	0.24	0.26	0.25	0.24	0.23	non	non
	**aW_2**	0.15	0.21	0.21	0.25	0.21	0.23	0.18	0.28	0.22	0.21	0.27	0.24	0.26	0.25	0.24	0.21	non	non
	**aW_3**	0.16	0.14	0.18	0.21	0.22	0.15	0.17	0.18	0.24	0.27	0.22	0.22	0.25	0.25	0.24	0.22	non	non
	**aW_4**	0.18	0.18	0.19	0.24	0.21	0.17	0.23	0.16	0.21	0.24	0.22	0.22	0.25	0.26	0.22	0.22	non	non
	**aD_1**	0.15	0.22	0.18	0.22	0.18	0.20	0.16	0.17	0.11	0.12	0.12	0.14	0.13	0.12	0.07	0.02	non	non
	**aD_2**	0.21	0.21	0.21	0.18	0.19	0.20	0.18	0.17	0.10	0.12	0.11	0.10	0.13	0.12	0.07	0.03	non	non
	**aD_3**	0.15	0.19	0.15	0.19	0.16	0.16	0.15	0.16	0.11	0.11	0.11	0.10	0.13	0.12	0.07	0.03	non	non
	**aD_4**	0.11	0.19	0.18	0.16	0.20	0.17	0.15	0.16	0.12	0.09	0.11	0.11	0.13	0.13	0.07	0.03	non	non
	**eW_1**	0.16	0.12	0.19	0.17	0.14	0.08	0.16	0.12	0.09	0.12	0.13	0.11	0.15	0.15	0.11	0.13	0.14	0.14
	**eW_2**	0.14	0.12	0.09	0.11	0.15	0.09	0.10	0.09	0.10	0.11	0.14	0.10	0.15	0.15	0.11	0.13	0.14	0.14
	**eW_3**	0.13	0.14	0.15	0.11	0.12	0.09	0.10	0.12	0.07	0.10	0.12	0.11	0.15	0.14	0.11	0.13	0.14	0.14
	**eW_4**	0.12	0.12	0.14	0.07	0.14	0.08	0.01	0.10	0.10	0.12	0.11	0.11	0.14	0.14	0.11	0.13	0.15	0.14
	**ed_1**	0.09	0.12	0.13	0.10	0.09	0.08	0.06	0.10	0.06	0.07	0.07	0.06	0.08	0.07	0.04	0.04	0.03	0.02
	**ed_2**	0.10	0.12	0.11	0.09	0.10	0.07	0.09	0.08	0.05	0.06	0.06	0.06	0.07	0.07	0.04	0.04	0.03	0.02
	**ed_3**	0.10	0.11	0.09	0.15	0.10	0.06	0.09	0.08	0.07	0.08	0.06	0.06	0.07	0.07	0.04	0.04	0.03	0.02
	**ed_4**	0.12	0.14	0.10	0.09	0.08	0.06	0.06	0.11	0.07	0.06	0.06	0.05	0.07	0.05	0.04	0.03	0.03	0.02
Wheat	**aW_1**	0.433	0.443	0.507	0.416	0.348	0.328	0.372	0.437	0.441	0.55	0.533	0.538	0.451	non	non	non	non	non
	**aW_2**	0.32	0.632	0.511	0.585	0.546	0.495	0.571	0.441	0.536	0.539	0.378	0.47	0.599	non	non	non	non	non
	**aW_3**	0.507	0.513	0.494	0.501	0.461	0.369	0.483	0.464	0.487	0.434	0.568	0.474	0.569	non	non	non	non	non
	**aW_4**	0.54	0.636	0.415	0.596	0.418	0.522	0.535	0.582	0.596	0.604	0.545	0.499	0.607	non	non	non	non	non
	**aD_1**	0.366	0.441	0.269	0.319	0.284	0.324	0.143	0.11	0.11	0.0849	0.0481	0.0238	0.0117	non	non	non	non	non
	**aD_2**	0.413	0.302	0.496	0.534	0.373	0.173	0.231	0.101	0.153	0.0782	0.0345	0.0269	0.0166	non	non	non	non	non
	**aD_3**	0.428	0.388	0.461	0.205	0.481	0.236	0.218	0.14	0.127	0.0754	0.0289	0.042	0.0341	non	non	non	non	non
	**aD_4**	0.522	0.522	0.417	0.329	0.333	0.247	0.212	0.171	0.255	0.122	0.0402	0.0581	0.0303	non	non	non	non	non
	**eW_1**	0.211	0.288	0.294	0.293	0.231	0.409	0.349	0.399	0.368	0.456	0.255	0.211	0.266	non	non	non	non	non
	**eW_2**	0.207	0.242	0.363	0.272	0.216	0.428	0.336	0.388	0.379	0.344	0.39	0.21	0.31	non	non	non	non	non
	**eW_3**	0.222	0.176	0.225	0.272	0.374	0.403	0.324	0.336	0.44	0.35	0.256	0.181	0.272	non	non	non	non	non
	**eW_4**	0.287	0.216	0.234	0.32	0.325	0.281	0.369	0.437	0.422	0.358	0.276	0.294	0.298	non	non	non	non	non
	**ed_1**	0.249	0.178	0.298	0.167	0.255	0.206	0.229	0.256	0.214	0.141	0.0648	0.0281	0.0216	non	non	non	non	non
	**ed_2**	0.223	0.242	0.296	0.187	0.227	0.21	0.194	0.235	0.175	0.154	0.156	0.0424	0.0253	non	non	non	non	non
	**ed_3**	0.316	0.184	0.285	0.241	0.18	0.238	0.259	0.289	0.222	0.18	0.173	0.0594	0.0347	non	non	non	non	non
	**ed_4**	0.287	0.325	0.276	0.255	0.259	0.378	0.233	0.302	0.149	0.0316	0.0365	0.0249	0.0143	non	non	non	non	non

Note: aw= 400 ppm & well-wateded; ad= 400 ppm & drought stressed; ew=800 ppm & well-watered; ed= 800 ppm & droughted stressed.

Raw data of related physiological parameters at different harvest time during progressive soil drying

	Treatment	1st havrst				2nd havrst				3rd harvest				4th havest				5th harvest			
Leaf [ABA] of maize (μg g^{-1})	400ppm,well watered	0.62	0.47	0.54	0.41	1.10	0.63	0.33	0.79	0.34	0.53	0.46	0.76	0.89	0.76	0.58	0.71	0.21	0.22	0.31	0.55
	800ppm,well watered	0.83	1.19	1.56	0.84	0.71	1.02	1.04	0.83	1.02	1.34	0.84	0.89	0.82	0.60	0.97	1.12	0.71	0.78	0.98	1.07
	400ppm, drought stressed	0.62	0.47	0.54	0.41	0.85	0.55	0.83	0.79	1.14	1.27	0.86	1.38	1.47	1.65	1.95	3.24	2.37	1.91	2.53	4.74
	800ppm, drought stressed	0.83	1.19	1.56	0.84	1.10	1.24	1.39	0.91	1.17	1.41	1.65	1.21	4.86	3.34	3.28	2.82	5.26	4.85	4.50	3.45
Leaf [ABA] of wheat (μg g^{-1})	400ppm,well watered	0.24	0.32	0.56	0.39	0.55	0.58	0.40	0.60	0.36	0.40	0.74	0.67	0.71	0.35	0.50	0.50	0.41	0.48	0.40	0.52
	800ppm,well watered	0.32	0.41	0.42	0.25	0.39	0.47	0.26	0.42	0.19	0.51	0.21	0.27	0.34	0.50	0.25	0.37	0.25	0.64	0.27	0.31
	400ppm, drought stressed	0.24	0.32	0.24	0.32	0.43	0.62	0.48	0.69	0.65	0.94	0.68	0.59	0.89	1.23	1.38	3.43	2.60	3.28	2.30	3.41
	800ppm, drought stressed	0.32	0.41	0.32	0.41	0.47	0.49	0.46	0.48	0.57	0.66	0.59	0.62	1.40	1.66	1.23	2.22	4.23	5.28	3.21	3.08
Biomss of maize (g pot^{-1})	400ppm,well watered	2.40	1.83	1.48	2.00	4.90	4.17	4.98	4.79	7.86	10.43	8.84	8.67	25.49	24.14	21.41	25.02	24.15	22.16	18.97	21.09
	800ppm,well watered	2.74	1.61	1.21	2.24	5.31	6.24	5.71	6.28	31.39	34.76	40.10	27.89	13.20	11.76	10.38	9.87	20.33	23.83	22.04	18.40
	400ppm, drought stressed	2.40	1.83	1.48	2.00	5.17	4.77	4.82	4.81	10.10	7.89	9.58	9.71	14.59	16.69	12.81	17.47	19.14	18.58	20.25	14.23
	800ppm, drought stressed	2.74	1.61	1.21	2.24	4.58	3.82	4.90	5.56	10.34	8.33	7.98	8.57	17.04	15.84	15.97	15.36	18.40	18.76	18.01	23.26
Biomss of wheat (g pot^{-1})	400ppm,well watered	5.10	5.06	5.84	4.85	6.64	6.85	6.64	6.75	8.52	8.79	8.73	9.82	12.36	12.68	12.58	10.05	14.78	13.95	15.00	15.22
	800ppm,well watered	5.62	5.57	5.29	6.96	8.64	8.37	8.14	9.83	11.46	12.99	10.98	13.85	12.84	13.30	17.21	15.48	19.56	16.61	17.24	16.91
	400ppm, drought stressed	5.10	5.06	5.84	4.85	6.51	7.24	6.33	7.57	10.42	9.09	9.26	8.69	8.46	11.99	12.97	11.55	14.25	14.16	13.46	13.92
	800ppm, drought stressed	5.62	5.57	5.29	6.96	7.86	7.26	8.36	8.78	10.37	9.97	10.06	11.54	16.50	13.52	11.60	13.05	16.14	17.99	18.31	15.76
Leaf area of maize (cm^2)	400ppm,well watered	338.88	313.47	410.31	394.35	862.83	746.22	841.78	854.48	1375.44	1528.40	1589.37	1546.86	3954.77	3465.82	3358.21	3543.28	3271.16	3084.27	2957.55	3524.18
	800ppm,well watered	470.57	391.81	316.97	493.71	947.22	1251.84	939.73	1116.71	3877.61	4458.33	4415.22	3751.01	1842.64	1856.31	1574.26	1314.13	3003.80	3931.11	3934.23	2749.58
	400ppm, drought stressed	338.88	313.47	410.31	394.35	898.89	748.70	854.51	912.61	1701.35	1344.42	1501.38	1450.19	1932.44	2158.37	1832.47	2485.11	2097.40	1987.21	2185.42	1840.33
	800ppm, drought stressed	470.57	391.81	316.97	493.71	831.07	637.41	883.36	951.34	1404.52	1404.52	1268.68	1488.22	2574.22	2542.81	2481.23	2283.45	2258.31	2279.05	2345.27	2597.18

**Leaf area of wheat (cm^2)**	400ppm,well watered	706.58	653.48	754.27	736.11	799.27	781.38	776.87	620.79	882.15	926.44	846.10	906.12	1136.72	1194.85	1305.28	931.83	1144.38	1047.38	1299.37	1107.05
	800ppm,well watered	815.11	861.35	789.44	912.31	972.31	1069.48	905.98	1066.87	1275.11	1372.57	1247.10	1388.68	1271.96	1194.59	1625.78	1526.85	1393.30	1214.13	1405.94	1598.32
	400ppm, drought stressed	823.31	641.37	875.14	887.22	838.10	754.56	786.98	735.44	1067.35	844.41	958.64	826.05	630.85	965.84	1053.11	948.47	568.34	479.55	632.41	539.41
	800ppm, drought stressed	878.11	857.21	888.24	785.52	770.66	849.35	897.13	1015.28	1103.28	1021.02	1011.84	1090.40	1386.41	1186.20	976.89	1113.18	595.41	782.28	550.56	892.33
**Leaf water potical of maize (Mpa)**	400ppm,well watered	4.60	5.80	5.60	6.00	5.00	5.40	4.40	4.90	5.20	6.10	5.20	5.20	6.40	7.00	6.20	6.40	6.40	6.50	6.80	6.20
	800ppm,well watered	6.40	5.40	6.20	5.80	5.80	5.50	6.20	5.60	5.00	4.80	4.40	4.50	4.80	5.20	4.90	4.80	5.60	6.00	4.60	5.20
	400ppm, drought stressed	4.60	5.80	5.60	6.00	5.20	5.60	5.80	5.00	7.10	6.20	5.80	5.50	9.30	9.80	8.90	9.80	14.40	15.60	16.20	15.10
	800ppm, drought stressed	6.40	5.40	6.20	5.80	7.00	6.80	6.30	6.80	5.80	5.20	5.60	5.20	7.80	7.40	7.80	8.30	9.40	10.20	11.20	10.40
**Leaf water potical of wheat (Mpa)**	400ppm,well watered	0.91	1.08	0.92	0.95	1.08	1.12	1.06	1.12	1.18	1.06	1.25	1.30	1.08	1.14	1.02	1.07	1.22	1.18	1.17	1.25
	800ppm,well watered	1.12	1.10	1.08	1.06	0.98	1.02	1.14	1.20	1.02	1.18	1.05	1.19	1.01	1.10	1.03	1.09	1.09	1.03	1.15	1.14
	400ppm, drought stressed	1.03	1.11	0.92	1.06	1.26	1.33	1.14	1.16	1.29	1.38	1.22	1.31	1.41	1.28	1.31	1.43	2.44	2.31	2.45	2.21
	800ppm, drought stressed	1.11	1.10	1.14	1.04	1.18	1.04	1.18	1.27	1.22	1.35	1.12	1.28	1.25	1.39	1.35	1.27	2.22	2.28	2.31	1.65
**Leaf relative water content of maize(%)**	400ppm,well watered	95.61	96.10	91.10	94.91	94.45	93.75	97.28	94.89	95.65	92.65	92.42	96.00	93.56	92.97	92.25	92.06	92.73	96.09	94.33	93.46
	800ppm,well watered	94.41	96.30	95.72	94.64	91.39	94.94	94.65	95.61	90.54	93.17	94.51	95.85	95.87	92.94	93.33	92.71	89.63	94.21	93.93	90.46
	400ppm, drought stressed	95.61	96.10	91.10	94.91	95.91	94.61	95.72	96.28	93.16	92.30	93.17	92.03	85.44	92.58	90.57	84.48	47.49	61.23	62.75	61.71
	800ppm, drought stressed	94.41	96.30	95.72	94.64	92.88	91.77	91.18	94.25	91.82	94.09	91.90	92.76	90.54	92.72	92.30	88.04	84.03	78.61	73.16	65.34
**Leaf relative water content of wheat(%)**	400ppm,well watered	85.78	89.32	86.67	88.15	89.62	91.59	89.37	89.53	89.33	88.51	86.98	90.06	93.37	93.94	93.77	95.62	90.75	90.64	90.65	92.26
	800ppm,well watered	87.19	89.91	92.25	87.18	90.95	93.18	93.26	88.70	90.97	92.41	90.86	92.20	93.98	95.42	94.80	95.98	95.83	96.58	96.12	95.34
	400ppm, drought stressed	87.10	86.87	86.73	90.73	88.24	88.61	92.03	85.04	78.87	83.41	87.94	85.54	70.53	72.71	71.27	72.41	54.59	45.34	47.84	47.48
	800ppm, drought stressed	88.12	85.45	88.44	88.77	90.26	92.02	88.72	91.95	91.43	92.42	91.10	92.29	89.86	85.80	86.68	88.65	73.61	57.99	65.65	63.09
